# T‐Cadherin in Biliary Tract Cancer Stroma, a Potent Pharmacological Target for Biliary Tract Carcinogenesis

**DOI:** 10.1002/cai2.70001

**Published:** 2025-02-28

**Authors:** Yuki Hanamatsu, Chiemi Saigo, Tamotsu Takeuchi

**Affiliations:** ^1^ Department of Pathology and Translational Research Gifu University School of Medicine Gifu Japan; ^2^ Center for One Medicine Innovative Translational Research; COMIT Gifu University Gifu Japan; ^3^ The United Graduate School of Drug Discovery and Medical Information Sciences Gifu Japan

**Keywords:** biliary tract cancer, cancer stroma, T‐cadherin

**Abbreviation**
BTC: biliary tract cancer

Based on the empirical data, we propose that T‐cadherin could be a molecular target for disrupting the stroma of patients with biliary tract cancer (BTC).

BTC comprises carcinomas originating in the bile ducts, including cholangiocarcinomas (cancers arising in the intrahepatic or extrahepatic bile ducts) and gallbladder carcinomas [1]. BTC often exhibits an aggressive clinicopathological course [1]. Surgical resection remains the most curative treatment option for patients with BTC; however, it may be limited to the early stages of cancer [1]. Owing to their poor sensitivity to chemotherapeutic agents, new therapeutic approaches are required for patients with advanced BTC.

One of the remarkable pathological features of BTC is the dense fibrous stroma harboring cancer cell nests. It is well established that stromal cells play a crucial role in the tumor microenvironment. Therefore, several targeting therapies are attempted against cancer stroma. For example, lysyl oxidases (LOXs) are a family of five secreted copper‐dependent amine oxidases (LOX and LOXL1–4) that promote carcinogenesis by generating cancer stroma. Very recently, Burchard et al. [2] demonstrated that PXS‐5505, which is a small molecule inhibitor of all LOX isoforms, improved chemotherapeutic penetration and reduced the inflammatory reaction of intrahepatic cholangiocarcinoma, thereby enhancing antitumor immunity in autochthonous and orthotopic murine models. Unfortunately, efforts to target individual LOX isoforms have failed to achieve clinical impact, likely due to the compensatory action of other LOX family members. Combination therapies targeting multiple stromal components are warranted.

T‐cadherin is an atypical cadherin attached to the plasma membrane by a glycosylphosphatidylinositol anchor without a cytosolic domain [2]. Notably, it is overexpressed in endothelial cells of tumor‐penetrating vessels in several malignant tumors [3, 4].

In this study, we investigated whether T‐cadherin was also expressed in the tumor endothelial cells of BTC. Immunohistochemical staining using a tissue microarray, with a core diameter of 1.5 mm, demonstrated T‐cadherin immunoreactivity in cancer stromal niches in BTC, especially in the cancer invasion microenvironment with a desmoplastic reaction (Figure 1a–d). Furthermore, T‐cadherin expression was detected in the endothelial cells of tumor vessels and stromal mesenchymal cells of all 27 intrahepatic cholangiocarcinomas and 32 of 43 extrahepatic biliary duct adenocarcinomas. Consistent with previous research [3], T‐cadherin immunoreactivity was also observed in the endothelial cells of tumor‐penetrating vessels in breast and colorectal cancers. However, little T‐cadherin immunoreactivity was observed in the stromal mesenchymal cells of these cancers (Figure 1e,f).

**Figure 1:**
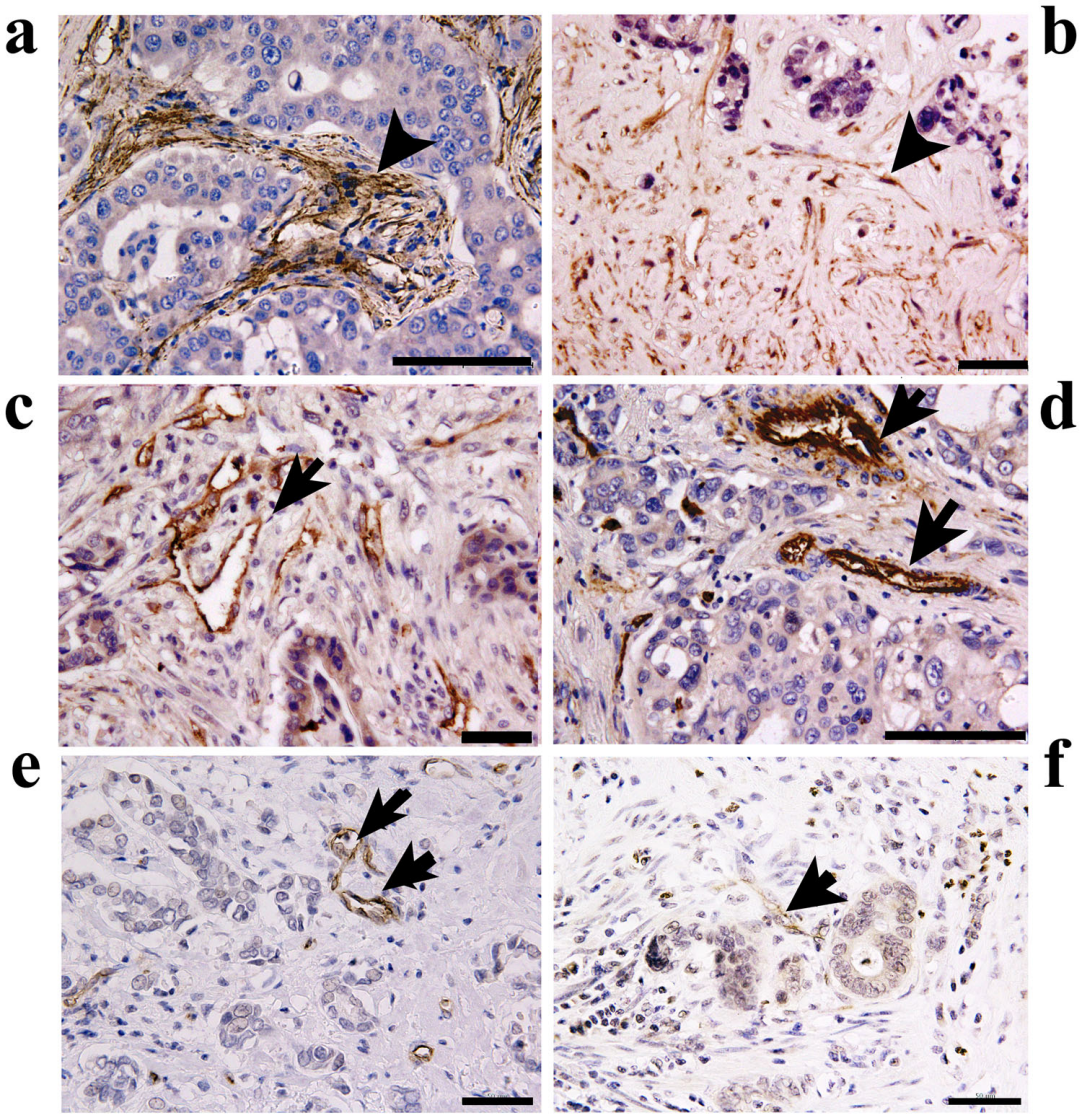
T‐cadherin immunoreactivity in BTC tissue specimens. T‐cadherin immunoreactivity was found in stromal mesenchymal cells at the cancer invasion front (a and b). Endothelial cells of tumor vessels exhibited strong T‐cadherin immunoreactivity (c and d). Notably, weak T‐cadherin immunoreactivity was observed in BTC cells in (b) and (c), whereas negligible T‐cadherin immunoreactivity was found in BTC cells in (a) and (d). T‐cadherin immunoreactivity was also found in endothelial cells of tumor vessels in breast cancer (e) and colorectal cancer (f). Contrastingly, stromal mesenchymal cells exhibit little T‐cadherin immunoreactivity in breast or colorectal cancer. Arrowhead and arrow indicate T‐cadherin immunoreactivity in tumor stromal mesenchymal and tumor vascular endothelial cells, respectively. Tissue microarray was purchased from US Biomax (Rockville, Maryland, the United States). This company obtained informed consent. The scale bar indicates 100 μm (a–d) and 50 μm (e and f).

Here, we could not unravel whether stromal T‐cadherin expression was related to clinicopathological features, including prognosis, resistance to therapy, or metastasis. Moreover, due to the intratumoral heterogeneity, subsequent studies exploring the pathobiological property of stromal expression of T‐cadherin using whole tissue specimens implementing various clinicopathological parameters are warranted.

Robust fibrous stroma, a characteristic feature of BTC, impedes immune cell penetration [5]. Strikingly, recent research has revealed that exosomal PD‐L1 confers immune evasion to cancer cells [6]. Consequently, immune checkpoint inhibitor therapy faces challenges such as a low response rate in BTC [7]. Several preclinical approaches apply an exosome inhibitor, GW4869, against cancer progression [8]. Notably, the adiponectin–T‐cadherin pathway also mediates exosome biogenesis [9]. We hypothesize that targeting T‐cadherin could be a novel therapeutic strategy that might reduce exosomal PD‐L1 and increase the effect of immune checkpoint inhibitors in BTC.

T‐cadherin null mice are viable and fertile [10], suggesting that other molecules could complement the physiological properties of T‐cadherin. We propose that targeting T‐cadherin could enhance the efficacy of immunotherapy in patients with BTC. Furthermore, combination therapies targeting multiple stromal components with PXS‐5505, GW4869, or more might be more effective.

## Author Contributions

**Yuki Hanamatsu:** funding acquisition (equal), investigation (equal), writing – original draft (equal). **Chiemi Saigo:** data curation (equal), investigation (supporting), writing – original draft (lead). **Tamotsu Takeuchi:** conceptualization (equal), data curation (equal), funding acquisition (equal), writing – review and editing (lead).

## Ethics Statement

The authors have nothing to report.

## Consent

The authors have nothing to report.

## Conflicts of Interest

The authors declare no conflicts of interest.

## Data Availability

Datasets generated and/or analyzed during the current study are available from the corresponding author upon reasonable request.
